# Cervical spine immobilization does not interfere with nasotracheal intubation performed using GlideScope videolaryngoscopy: a randomized equivalence trial

**DOI:** 10.1038/s41598-022-08035-0

**Published:** 2022-03-08

**Authors:** Yi-Min Kuo, Hsien-Yung Lai, Elise Chia-Hui Tan, Yi-Shiuan Li, Ting-Yun Chiang, Shiang-Suo Huang, Wen-Cheng Huang, Ya-Chun Chu

**Affiliations:** 1 Department of Anesthesiology, Taipei Veterans General Hospital, Beitou Dist., Taipei, 11217 Taiwan; 2 School of Medicine, National Yang Ming Chiao Tung University, Hsinchu, 30010 Taiwan; 3 Department of Anesthesiology, Mennonite Christian Hospital, Hualien, 970 Taiwan; 4 National Research Institute of Chinese Medicine, Ministry of Health and Welfare, Taipei, 11217 Taiwan; 5 Institute of Hospital and Health Care Administration, National Yang Ming Chiao Tung University, Hsinchu, 30010 Taiwan; 6 Department of Pharmacology, Institute of Medicine, Chung Shan Medical University, Taichung, 40201 Taiwan; 7 Department of Pharmacy, Chung Shan Medical University Hospital, Taichung, 40201 Taiwan; 8 Department of Neurosurgery, Neurological Institute, Taipei Veterans General Hospital, Taipei, 11217 Taiwan

**Keywords:** Medical research, Risk factors

## Abstract

GlideScope-assisted nasotracheal intubation (NTI) has been proposed as an alternative to difficult orotracheal intubation for critical patients or those under cervical immobilization. We evaluated the difficulty of performing NTI using GlideScope under cervical orthosis. A total of 170 patients scheduled for elective cervical spinal surgery that required NTI were randomized to receive cervical immobilization using a cervical collar (collar group) or no cervical immobilization at all (control group) before anesthetic induction (group assignment at 1:1 ratio). All NTI during anesthetic induction were performed using the GlideScope. The primary outcome was time to intubation. The secondary outcomes were ease of intubation, including the necessity of auxiliary manipulations to assist intubation, and the nasotracheal intubation difficulty scale (nasoIDS). An exploratory analysis identified morphometric parameters as predictors of time to intubation, the necessity of auxiliary manipulations, and a nasoIDS score ≥ 4. For time to intubation, the mean difference (collar group—control) was − 4.19 s, with a 95% confidence interval (CI) of − 13.9 to 5.52 that lay within our defined equivalence margin of 16 s. Multivariate regressions precluded the association of cervical immobilization with a necessity for auxiliary manipulations (adjusted odds ratio [aOR] 0.53, 95% CI [0.26–1.09], *P* = 0.083) and a nasoIDS ≥ 4 (aOR 0.94 [0.84–1.05], *P* = 0.280). Among all morphometric parameters, the upper lip bite test class was predictive of a longer time to intubation (all analyses relative to class 1, 14 s longer for class 2, *P* = 0.032; 24 s longer for class 3, *P* = 0.070), increased necessity for auxiliary manipulation (aOR 2.29 [1.06–4.94], *P* = 0.036 for class 2; aOR 6.12 [1.04–39.94], *P* = 0.045 for class 3), and nasoIDS ≥ 4 (aOR 1.46 [1.14–1.89], *P* = 0.003 for class 3).The present study demonstrated that GlideScope achieved NTI in patients with or without cervical immobilization equivalently with respect to intubation time and ease.

## Introduction

Cervical immobilization increases the difficulty of airway management by narrowing the mouth opening, reducing the angles of neck extension, and obscuring the glottis during laryngoscopy^1–4^. A video laryngoscope is recommended as an alternative device in airway management for cervical spine surgeries and patients under cervical immobilization^5–7^. The video laryngoscope requires less neck movement to achieve a view of the glottis compared with the conventional direct laryngoscopy (DL) technique^8–10^, is less technically demanding than a fiberoptic bronchoscope, and has a high first-attempt success rate^6,7^. Additionally, videolaryngoscope helps the clinician obtain a modified Cormack–Lehane (MCL) grade 1 view and reduces oropharyngeal complications during tracheal intubation^6^.

Nasotracheal intubation (NTI), which is frequently used for oral and maxillofacial surgery^11^, is sometimes used in surgeries of the neck region to facilitate surgical positioning and decrease the likelihood of oropharyngeal complications. It is preferred by some spine surgeons and used to lessen the pressure from the oral tube which causes compression injury of tissues during manipulations or surgical retraction at the upper neck region of anterior cervical spine surgeries. NTI may be the primary choice of intubation because of its favorable airway-related outcomes following anterior cervical spine surgery^12–14^. The GlideScope video laryngoscope has been deemed to be an ideal intubation device for NTI^15,16^. GlideScope-assisted NTI was reported an alternative to difficult orotracheal intubation for critical patients in the intensive care unit^17^ or under cervical orthosis^18^. However, whether cervical collars, which are frequently used among patients with myelopathy to prevent possible harm during laryngoscopy or transfer, impede NTI during anesthetic induction remain unanswered.

To date, video laryngoscopy is the most commonly used technique for initial intubation in patients with unstable cervical spine or those under cervical spine immobilization^19,20^. Most studies on this topic have employed orotracheal intubation experiments^7^, and their results poorly elucidate NTI in patients with restricted neck movement. The study examined the difficulty of GlideScope-assisted NTI under cervical immobilization. Considering the difficulty of NTI under cervical immobilization, we conducted a randomized equivalence study. Our primary aim was to determine whether time to intubation (the time for the nasotube to advance from the oropharynx into the trachea) was prolonged in patients with cervical immobilization. Secondarily, we determined the ease of NTI through the analysis of the necessity of auxiliary manipulations to assist intubation and the nasotracheal intubation difficulty scale (nasoIDS)^21^ between the experimental and control groups.

## Results

### Study population

**Figure 1:**
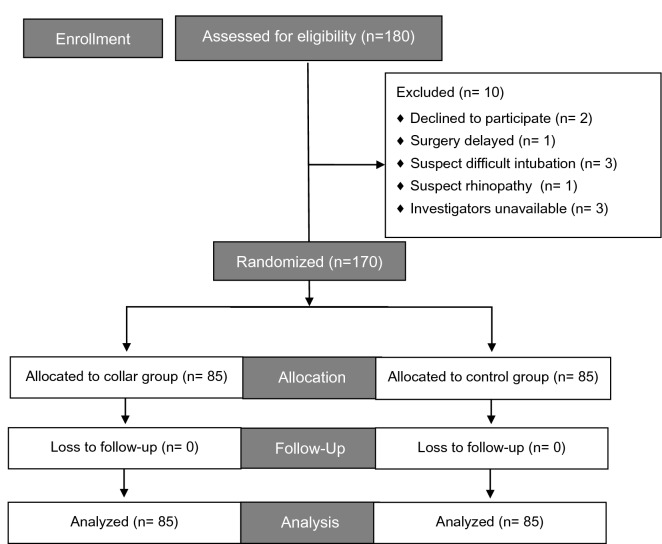
Consort diagram describing patient enrollment.

A total of 170 patients were randomly allocated to two groups, with 85 patients in each group. All 170 patients completed the study. The CONSORT diagram of patient enrollment is presented in Fig. 1. No difference was observed between the two groups in patient characteristics (Table 1). All NTIs were successfully performed, and no patient required unfastening of the cervical collar to facilitate intubation during the procedure.

**Table 1:** Patient characteristics.

Variables	Collar (n = 85)	Control (n = 85)	*P*
Age (year)	58 (50–68)	59 (51–68.5)	0.533
Sex (male)	51 (60.0)	45 (52.9)	0.439
BMI (kg m^−2^)	25.7 (22.8–28.0)	25.1 (22.8–27.1)	0.623
Neck scar or goiter (%)	4 (4.7)	3 (3.5)	1.000
Inter-incisor gap (cm)	3.7 (3.3–4.0)	3.8 (3.4–4.2)	0.791
Thyromental distance (cm)	8.5 (8.0–9.0)	9 (8.0–9.8)	0.141
Sternomental distance (cm)	15.5 (14.0–16.8)	16 (14.0–17.0)	0.361
Ratio of height to thyromental distance	18.9 (17.5–20.5)	18.2 (17.2–19.9)	0.244
Neck circumstance (cm)	39 (37.0–41.0)	37.6 (35.0–40.3)	0.114
**Modified Mallampati test**			0.442
Class 1	19 (22.4)	21 (24.7)	
Class 2	43 (50.6)	48 (56.5)	
Class 3	23 (27.1)	16 (18.8)	
Class 4	0	0	
**Upper lip bite test**			0.113
Class 1	60 (70.6)	47 (55.3)	
Class 2	21 (24.7)	33 (38.8)	
Class 3	4 (4.7)	5 (5.9)	
**Range of motion of the atlanto–occipital joint**			0.123
Class 1 (> 35°)	53 (62.4)	65 (76.5)	
Class 2 (22°–34°)	30 (35.3)	15 (17.6)	
Class 3 (12°–21°)	2 (2.4)	5 (5.9)	

Data are presented as median (interquartile range) or number (%).

### Intubation time and ease of NTI

**Figure 2:**
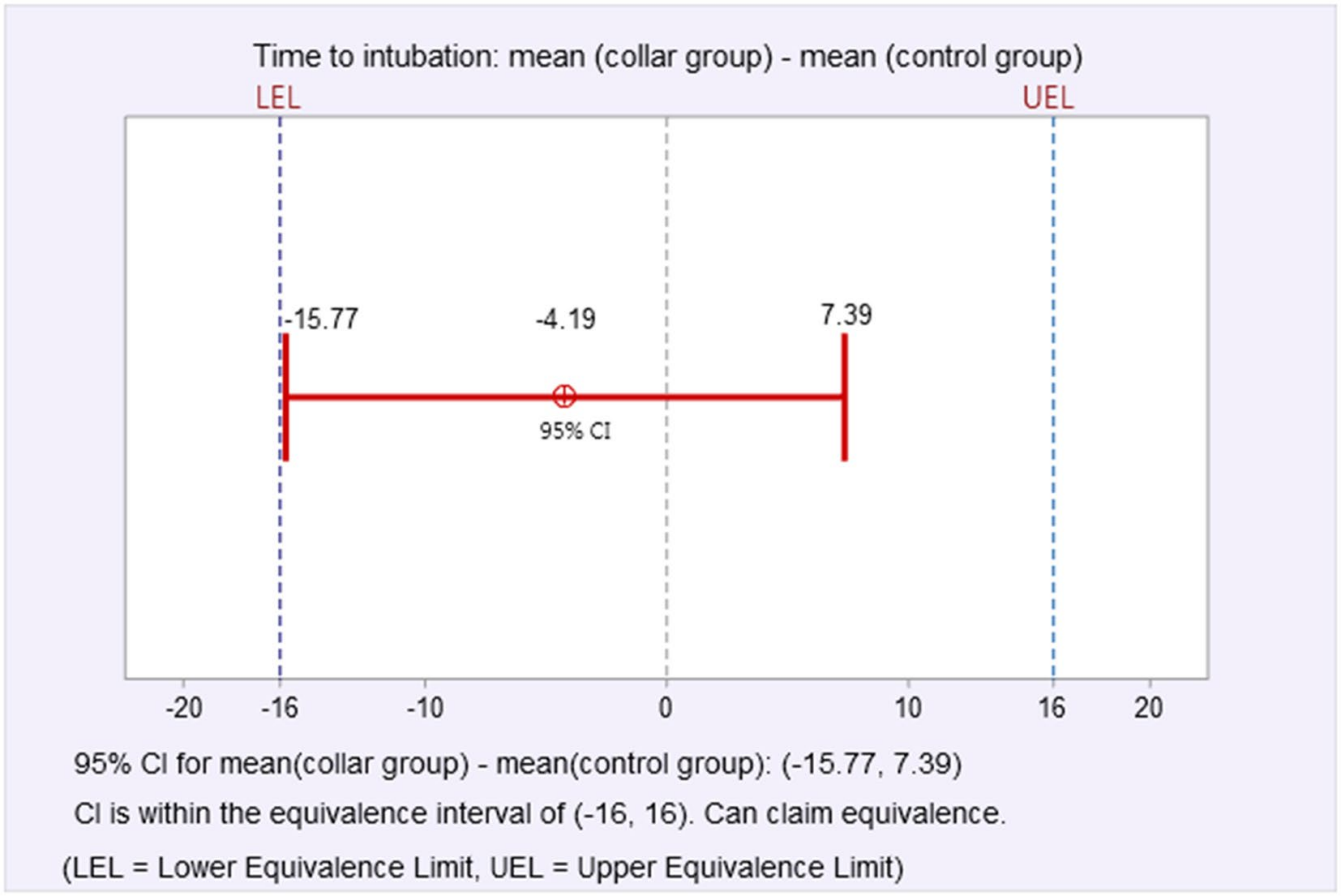
Mean difference (90% confidence interval) of time to intubation and results of the equivalence test of means after two one-sided tests.

The primary outcome of the mean difference of time to intubation between groups was − 4.19 s (mean of collar group − mean of control group). The upper and lower bounds of the 90% CI were 5.52 s and − 13.9 s, respectively, and lay within our defined equivalence time difference of 16 s; clinical equivalence was inferred accordingly (Fig. 2). The secondary outcomes of time for nasal passage, time for glidescopy, total time for NTI, POGO score, and MCL grading did not differ significantly between the groups (Table 2). Less NTIs of the collar group required auxiliary manipulations during tube passage from the oropharynx into the trachea (31.8% vs. 48.2% in control group, *P* = 0.041; Table 2). Effective auxiliary manipulations to successfully assist intubation did not differ across method types, with the exception of the cuff inflation method that significantly facilitated NTI in more control group patients (7.1% in the collar group vs. 18.8% in the control, *P* = 0.038, Table 2).

**Table 2:** Intubation outcomes.

Intubation outcomes	Collar (n = 85)	Control (n = 85)	*P*
**Total time for NTI (s)**	30 (23.5–43)	31 (24–51)	0.387
Time for nasal passage	10 (9–13)	12 (9–15)	0.543
Time for glidescopy	6 (5–8)	5 (4–7)	0.308
Time to intubation	10 (7–21)	13 (8–30.5)	0.156
**NasoIDS score**			0.402
≤ 3	72 (84.7)	67 (78.8)	
4	9 (10.6)	15 (17.6)	
5	4 (4.7)	3 (3.5)	
6	0	0	
POGO	100 (100–100)	100 (100–100)	0.119
MCL grading			0.149
1	68 (80.0)	75 (88.2)	
2a and 2b	16 (18.8)	9 (10.6)	
3	1 (1.2)	1 (1.2)	
4	0	0	
Necessity of auxiliary manipulations	27 (31.8)	41 (48.2)	0.041
**Effective manipulations to complete NTI**			
Anterior laryngeal pressure	12 (14.1)	17 (20.0)	0.415
Cuff inflation	6 (7.1)	16 (18.8)	0.038
Change position of head	2 (2.4)	2 (2.4)	1.000
Magill forceps	6 (7.1)	3 (3.5)	0.496
Two/more concomitant manipulations	1 (1.2)	3 (3.5)	0.621
Change of heart rate after intubation (beats/min)*	+ 2.0 (− 2.0–6.0)	+ 3.0 (− 1.0–10.0)	0.266
Change of mean blood pressure after intubation (mmHg)*	+ 4.0 (− 4.3–16.0)	+ 12.0 (− 0.2–21.7)	0.016

Data are presented as median (interquartile range) or number (%).

*NTI* nasotracheal intubation, *NasoIDS* nasotracheal intubation difficulty scale, *POGO* percentage of glottic opening, *MCL* modified Cormack–Lehane, *Nasal preparation* tracheal tube passing through the nose to the oropharynx, *glidescopy* from insertion of the GlideSope to visualization of the glottis, *Intubation* tracheal tube advancing from the oropharynx into the trachea.

*2 min after tracheal intubation.

### Factors predicting the difficulty of GlideScope-assisted NTI

Multivariate regressions with GEE modeling precluded the association of cervical collars with time to intubation (*P* = 0.452), the necessity of auxiliary manipulations (aOR 0.53, 95% CI [0.26–1.09], *P* = 0.083), and nasoIDS ≥ 4 (aOR 0.94 [0.84–1.05], *P* = 0.280; Table 3). Among all morphometric parameters, the upper lip bit test (ULBT)^22^ class was predictive of intubation outcomes during NTI. Patients of ULBT class 2 had a 14 s longer for time to intubation (*P* = 0.032 vs. class 1), and that time for ULBT class 3 was 24 s longer (*P* = 0.070, vs. class 1; Table 3). ULBT class was also a predictor of the necessity for auxiliary manipulation (relative to class 1, aOR 2.29 [1.06–4.94], *P* = 0.036 for ULBT class 2; aOR 6.12 [1.04–39.94], *P* = 0.045 for ULBT class 3; Table 3). ULBT class 3 was predictive of nasoIDS ≥ 4 (aOR 1.46 [1.14–1.89], *P* = 0.003 vs. class 1; Table 3). The other factor predictive of nasoIDS ≥ 4 was the presence of neck scar or mass lesions (aOR 1.51 [1.14–2.00], *P* = 0.005; Table 3).

**Table 3:** Summary of multivariate analysis with generalized estimating equation modeling for variables associated with (1) time to intubation, (2) the necessity of auxiliary manipulations, and (3) nasoIDS ≥ 4 during NTI.

	y = time to intubation			y = necessity of auxiliary manipulations (vs. no necessity)		y = NasoIDS ≥ 4 (vs. < 4)	
	Estimate (beta)	SE	*P*	Adjusted OR (95% CI)	*P*	Adjusted OR (95% CI)	*P*
Intercept	− 64.31	97.20	0.508				
Cervical collar (Ref = control)	− 4.48	5.97	0.452	0.53 (0.26–1.09)	0.083	0.94 (0.84–1.05)	0.280
Age	− 0.16	0.27	0.550	1.05 (1.01–1.09)	0.007	1.00 (1.00–1.01)	0.716
Male (Ref = female)	− 10.06	7.90	0.203	0.69 (0.24–1.98)	0.493	0.98 (0.84–1.14)	0.761
BMI	0.31	0.85	0.710	0.99 (0.88–1.10)	0.798	1.00 (0.94–1.02)	0.958
Neck scar/mass lesion	5.54	14.96	0.711	2.55 (0.41–15.8)	0.314	1.51 (1.14–2.00)	0.005
Inter-incisor gap	3.08	5.55	0.579	0.75 (0.37–1.51)	0.426	0.95 (0.85–1.05)	0.312
Thyromental distance	− 1.97	6.24	0.752	0.58 (0.25–1.37)	0.215	0.91 (0.80–1.02)	0.1
Sternomental distance	2.71	2.79	0.331	1.52 (1.01–2.28)	0.044	1.05 (1.00–1.11)	0.062
Ratio of height to thyromental distance	1.02	2.49	0.683	0.89 (0.65–1.23)	0.495	0.99 (0.95–1.04)	0.797
Neck circumference	0.75	0.71	0.290	1.07 (0.94–1.21)	0.310	1.00 (0.99–1.02)	0.511
**Modified Mallampati test (Ref = Class 1)**							
Class 2	9.64	7.52	0.200	1.04 (0.42–2.56)	0.936	1.07 (0.93–1.24)	0.333
Class 3	14.73	8.78	0.093	1.08 (0.38–3.08)	0.886	1.09 (0.92–1.29)	0.314
**Upper lip bite test (Ref = Class 1)**							
Class 2	14.07	6.55	0.032	2.29 (1.06–4.94)	0.036	1.11 (0.98–1.26)	0.095
Class 3	24.33	13.43	0.070	6.12 (1.04–39.94)	0.045	1.46 (1.14–1.89)	0.003
**Range of motion of the atlanto–occipital joint (Ref = Class 1 > 35°)**							
Class 2 (22°–34°)	2.12	7.40	0.775	0.97 (0.40–2.38)	0.948	0.95 (0.82–1.09)	0.467
Class 3 (12°–21°)	− 21.91	17.23	0.204	1.15 (0.13–9.84)	0.900	0.73 (0.53–1.02)	0.061

*nasoIDS* nasotracheal intubation difficulty scale.

## Discussion

The result of this study was that GlideScope achieved equivalent times for successful NTI in patients with or without cervical immobilization. The multivariate analysis demonstrated that the presence of a cervical collar does not hinder GlideScope-assisted NTI with respect to time to intubation, nasoIDS, and the necessity for auxiliary manipulations to assist intubation. Our results provide evidence regarding GlideScope-assisted NTI in patients under cervical immobilization, and fill the knowledge gap regarding the use of GlideScope for airway management in this patient population.

The successful alignment of the tip of the nasotube with the glottic opening may underlie the easier passage of the nasotube into the trachea during the GlideScope-assisted NTI^15,16^. The alignment is unaffected by the use of cervical immobilization according to our results. The advantage is likely a result of the hypercurved blade design (hyperangulated blade of 60°) of the GlideScope. The more curved the blade, the higher the POGO score^23,24^, the less lifting force required for video laryngoscopy, and the less demand to lift, flex, or extend the neck to achieve a glottic view. Thus, the upper airway was less distorted^25–27^, and the nasotube could pass into the trachea following the natural trajectory as during a successful blind NTI without auxiliary manipulations. Accordingly, GlideScope-assisted NTI may serve an effective and safe alternative for airway management for patients who underwent cervical spine surgeries and are immobilized using a cervical collar. Oral edema, a macroglossia, and partial edentulousness along with a concomitant cervical orthosis for immobilization may render the oral passage of the endotracheal tube to be at a sharp angle and impractical for entering the larynx. Consequently, GlideScope-assisted NTI has been proposed as an alternative to difficult orotracheal intubation for patients in an intensive care unit^17^ or under cervical orthosis^18^. However, our results do not necessarily apply to all types of video laryngoscopies. In a comparison of videolaryngoscopies used for NTI under cervical immobilization, the POGO score was higher and intubation was completed more rapidly using a C-MAC D-Blade videolaryngoscope (extra-curved blade of 40°; Karl Storz, Tuttlingen, Germany) than with a McCoy videolaryngoscope (standard curved blades with a modified hinged tip at the end of the blade; Optima, Timesco Ltd., London., England)^24^, indicating that blade angulation differentially affects NTI outcomes. The McGrath MAC laryngoscope also possesses hyperangulated blade of 60° and facilitated routine NTI^28^. It can also play a role for NTI under cervical immobilization and this require further investigation.

Our exploratory analysis indicated that ULBT class is predictive for GlideScope-assisted NTI; patients presenting with ULBT class 2 or 3 require more time to intubate, require auxiliary manipulation to assist intubation, and have a higher chance of experiencing intubation difficulty compared with patients of ULBT class 1. Morphometric characteristics, such as a Mallampati class 3 or 4 or a thyromental distance of less than 6 cm, can predict the difficulty of laryngoscopy during cervical spine immobilization^29^. No defined test has been identified for NTI. The ULBT was promoted as a method for screening the difficulty of DL-assisted orotracheal intubation^30^. The test evaluates the range and freedom of mandibular movement and dental architecture^30^, and it was reportedly more accurate than the Mallampati test and furnished more reliable predictions for difficult oral intubation using conventional DL^22,30–32^. Our results demonstrated that ULBT class is associated with prolonged time to intubation, a higher nasoIDS score, and a higher necessity of auxiliary manipulation to assist intubation. Nevertheless, our patients with a high ULBT class did not perceptibly demonstrate retrognathia, mandibular protrusion, or limited temporomandibular joint movement. We therefore posit that (1) a high ULBT class is associated with anatomical misalignment between the nasotube and the glottic opening and (2) ULBT serves as a workable test during preoperative evaluation.

This study has some limitations. First, the study did not include a comparison with DL. The use of a cervical collar is well known to increase the incidence of MCL grade 3 or 4 glottic views in conventional DL, and glottic views were significantly improved when the GlideScope was used^33,34^. This finding illustrates obtaining a clear glottis view through DL under cervical spine immobilization becomes extremely difficult. Thus, DL-assisted NTI may impose an unnecessary risk of intubation failure or potential neurological injury from vigorous or multiple attempts of laryngoscopy^10^. Therefore, no DL-assisted intubation group was established for comparison in our study. Second, our study comprised patients scheduled for elective cervical spine surgery without obvious neck deformities or tumors. The potential presence of intraoral tumors, hypopharyngeal malignancy, or extensive presentation of oral/neck scarring or stricture could alter the intubation success rate of GlideScope^35^. Hence, the results may not be applicable to patients with concomitant oral and laryngeal pathologies other than degenerative cervical spine disease. Finally, we did not perform morphometric tests, such as ULBT, after the application of the cervical collar. Studies have reported that the inter-incisor distance was considerably reduced by the application of cervical collars^2,33^, but ULBT class has not been mentioned. Therefore, no research has ascertained the effect of cervical collars on ULBT class. However, if cervical immobilization increases ULBT class, it leads to intubation difficulty in the collar group. Thus, ULBT class after collar immobilization may be irrelevant to intubation outcomes.

In conclusion, GlideScope achieved NTI in patients with or without cervical immobilization equivalently with respect to intubation time and ease. Among morphometric parameters, ULBT class is associated with the difficulty of GlideScope-assisted NTI and is useful for preoperative evaluation.

## Methods

### Ethics

This randomized parallel group assessor-blinded trial was conducted at Taipei Veterans General Hospital. The study protocol and all amendments were approved by the Institutional Review Board, Taipei Veterans General Hospital (IRB No.: 2017-06-009B, date of approval: 05/07/2017) and registered at ClinicalTrials.gov (registration number: NCT03210922, principal investigator: W-C Huang, date of registration: 07/07/2017) prior to patient enrollment. Written informed consent was obtained from all patients before randomization. The study was conducted between October 2017 and October 2018, and performed in accordance with relevant guidelines and the Declaration of Helsinki. The reporting accords with the 2010 CONsolidated Standards of Reporting Trials (CONSORT). No changes to methods and definitions were made during this trial.

### Participants

This prospective study included patients aged 20–80 years of American Society of Anesthesiologists’ physical status I–III who were undergoing elective anterior cervical spine surgery and scheduled to receive general anesthesia with NTI as requested by the surgeon. Patients at risk of pulmonary aspiration of gastric contents, with abnormal coagulation function, with pathology of the nasal cavity, and an unstable cervical spine; those scheduled for fiberoptic tracheal intubation; and those who refused to provide their informed consent were excluded. The patients were examined at the preanesthetic visit; scores were recorded for the modified Mallampati test and mandibular protrusion in the upper lip bite test (ULBT)^30^; and measurements were taken for the inter-incisor distance during mouth opening, for the thyromental and sternomental distance upon neck extension, for the ratio of height to thyromental distance, for the range of motion of the atlanto–occipital joint, and for neck circumference. A single investigator evaluated all patients before surgery.

### Randomization and blinding

The enrolled patients were randomly assigned to receive (the collar group) or not receive (the control group) the cervical collar at a 1:1 ratio. A statistician prepared the randomization schedule and a research assistant prepared the random allocation sequence and kept them in an opaque, sealed envelopes. The co-investigator anesthesiologist enrolled the patients. Upon patient arrival to the operating room, the research assistance opened the envelope containing randomization information. The patients in the collar group were instructed to wear a cervical collar (Miami J Select, Össur, Reykjavik, Iceland), and patients in the control group were not instructed to wear the collar. Because the evaluator was present in the operating room during the intervention, effective blinding was not possible. The nasoIDS and use of auxiliary manipulation was scored by the anesthesiologist who performed tracheal intubation. All tracheal intubations were performed by a single anesthesiologist with more than 10 years of experience. Other outcomes, such as the time to intubation, the percentage of glottic opening (POGO), and MCL grade, were determined based on videos recorded from GlideScope monitors by a single assessor who was blinded to the study grouping.

### Study interventions

In the operating room, all patients underwent standard monitoring procedures. All patients were preoxygenated for 3 min. Anesthetic induction was standardized using propofol 1.5–2.5 mg kg^−1^, fentanyl 3 µg kg^−1^, and cis-atracurium 0.15 mg kg^−1^. The nares of the patients were then decongested and lubricated with mesh soaked in adrenaline (1/200,000). The tracheal tube Mallinckrodt Nasal Tracheal Tube (Covidien, Nakhon-Pathom, Thailand) was warmed and lubricated with a sterile water-soluble lubricant before use. The procedure and time for NTI was spilt into the following sections that were measured by an independent observer: (1) nasal passage, specifically the tracheal tube passing through the nose to the oropharynx; (2) glidescopy, specifically the insertion of GlideScope for a view of the glottis; and (3) intubation, specifically the tracheal tube advancing from the oropharynx into the trachea. The operator could use anterior laryngeal pressure to optimize the glottic view during the glidescopy. Should intubation be difficult, the operators used auxiliary manipulations, including anterior laryngeal pressure, cuff inflation^36^, head position adjustment, and the use of Magill forceps. A failed attempt was defined as any evidence of oxygen desaturation (peripheral O_2_ saturation < 90%), if the blade of the GlideScope was withdrawn from the mouth or if the nasotube was withdrawn from the nostril to recommence mask ventilation. The intubation procedure was recorded using the GlideScope Titanium Reusable System with LoPro blade (GlideScope Video Monitor; Verathon Medical, Burnaby, B.C., Canada). After tracheal intubation, patients were checked for possible complications, such as dental trauma, mucosal trauma, or epistaxis by the nurse anesthetist.

All nasotracheal tubes were removed from the patients after they fully recovered and met standard extubation criteria after the surgery. On the first postoperative day (POD1), the patient was followed-up for the presence of postoperative sore throat, nasal bleeding, and soft tissue laceration.

### Outcome measures

The primary outcome was time to intubation (tracheal tube advancing from the oropharynx into the trachea). The secondary outcomes were as follows: The first was ease of NTI, specifically the necessity of auxiliary manipulations to assist intubation and nasoIDS. The time to intubation and nasoIDS were scored from the video recorded by an investigator blinded to the study grouping. The nasoIDS was scored on an ordinal 6-point scale; 1 (*very easy*) = successfully intubated within 15 s on the first attempt; 2 (*easy*) = successfully intubated between 15 and 30 s on the first attempt; 3 (*moderate*) = successfully intubated after more than 30 s on the first attempt; 4 (*difficult*) = successfully intubated on the second attempt; 5 (*very difficult*) = successfully intubated on the third attempt; 6 (*unsuccessful*) = intubation could not be achieved within three attempts^21^. A high nasoIDS score (≥ 4) indicated an NTI that was relatively difficult, that required multiple attempts, or that was unsuccessful. The second secondary outcome comprised other intubation-related outcomes specifically time for nasal passage and GlideScopy, POGO score, MCL grading, and effective auxiliary manipulation that successfully assisted with intubation. The third secondary outcome comprised morphometric factors predicting (i) time to intubation, the (ii) necessity of auxiliary manipulations, and a (iii) nasoIDS score ≥ 4.

### Statistical analysis

Based on our pilot study of 20 patients who received NTI, the mean time to intubation was 29.6 ± 40.1 s in patients with cervical collar immobilization (*n* = 10) and 25.6 ± 21.5 s in control patients (*n* = 10). The mean times to intubation were 41.6 ± 40.0 s for patients who required auxiliary manipulation during intubation (*n* = 10), and 13.6 ± 6.7 s for patients who did not require this (*n* = 10). Therefore, we expected a prolonged mean time to intubation to be approximately 50% under cervical immobilization, and, therefore, the time approximate the mean time to intubation of those who required the auxiliary manipulations to assist intubation. Thereby we defined an equivalence margin of 16 s for time to intubation (41.6–25.6 s) as the range for clinical indifference. We expected the mean difference of time to intubation to be 5 s between the two groups, and the common standard deviation (SD) to be 21.5. An equivalence test of the means using two one-sided tests (TOST) and a parallel group design was performed to test the null hypothesis H_0_: μ2 − μ1 ≥ 16 or μ2 − μ1 ≤  − 16, versus the alternative hypothesis H_1_: − 16 <  μ2 − μ1 < 16. We estimated that 67 individuals per group (134 in total) must be randomized to reliably test our hypothesis at α = 0.05 and a 90% power. To account for a 10% of incomplete data rate or participants being lost to follow-up, we planned a target sample size of 85 patients per group (170 in total)^37^. Power analysis was performed using Minitab version 19 (State College, PA, USA).

The primary outcome of time to intubation was assessed for equivalence using the TOST method. If the upper and lower bounds of the 90% confidence interval (CI) were both less than the defined equivalence margin of 16 s, the criteria for equivalence between groups was met. For the secondary outcomes, the Shapiro–Wilk and Anderson–Darling tests were used to test the assumption of normality (*P* > 0.1). Normally distributed data are presented as the mean ± SD and were analyzed using an independent *t* test for unequal variances. Non-normally distributed interval and ordinal data were reported as the median (interquartile range [IQR]) and compared among groups using the Mann–Whitney–U test. Categorical variables were presented as their frequencies and evaluated using a χ^2^ or Fisher’s exact test in the case of low expected cell counts. The association with time to intubation was analyzed using generalized estimating equation (GEE) models with a normal distribution, and association with the necessity of auxiliary manipulations (vs. no such necessity) and the nasoIDS score ≥ 4 (vs. < 4), were estimated using GEE models with a binomial distribution. After multivariate adjustments, the standard error of the parameter estimate or adjusted odds ratios (aOR) with 95% CIs were reported, as appropriate. Statistical analyses were conducted using SAS version 9.4 for Windows (SAS, Cary, NC, USA), and a two-sided *P* value < 0.05 was considered statistically significant.
